# Critical Care–Based Decision‐Making and Timing of Pregnancy Termination in Hypertriglyceridemia‐Induced Acute Pancreatitis Complicated by Diabetic Ketoacidosis and Intrauterine Fetal Demise: A Case Report

**DOI:** 10.1155/crcc/4122751

**Published:** 2026-08-17

**Authors:** Yingshan Hu, Yue Mao, Hongmei Gao

**Affiliations:** ^1^ Department of Critical Care Medicine, Key Laboratory for Critical Care Medicine of the Ministry of Health, Emergency Medicine Research Institute, Tianjin First Central Hospital, Tianjin, China, tj-fch.com

**Keywords:** diabetic ketoacidosis, hypertriglyceridemia-induced acute pancreatitis, intrauterine fetal demise, plasma exchange, pregnancy

## Abstract

**Background:**

Acute abdominal pain accompanied by severe metabolic acidosis during pregnancy is frequently attributed to pregnancy‐related conditions. Hypertriglyceridemia‐induced acute pancreatitis (HTG‐AP) complicated by diabetic ketoacidosis (DKA) is rare in pregnancy and may be overlooked because of overlapping clinical and laboratory features.

**Case Presentation:**

We report a critically ill pregnant woman in the late second trimester who presented with abdominal pain and vomiting. Initial evaluation revealed severe metabolic acidosis and marked hypertriglyceridemia, and the patient was ultimately diagnosed with HTG‐AP complicated by DKA. During the disease course, with confirmed intrauterine fetal demise (IUFD), management goals transitioned to maternal‐centered critical care. However, the patient also exhibited profound metabolic derangement, intra‐abdominal hypertension, and systemic stress responses, rendering immediate pregnancy termination associated with a high perioperative risk. The patient was admitted to the intensive care unit (ICU) and received continuous renal replacement therapy (CRRT) to correct refractory metabolic acidosis, followed by therapeutic plasma exchange to rapidly reduce triglyceride burden. These interventions stabilized the internal milieu and served as a bridging strategy for definitive critical care decision‐making. After multidisciplinary evaluation, cesarean delivery was performed once maternal metabolic status had sufficiently stabilized. The patient′s condition gradually improved, and she was discharged in good recovery.

**Conclusions:**

This case highlights the diagnostic pitfalls of HTG‐AP complicated by DKA during pregnancy and underscores the importance of individualized decision‐making regarding the timing of pregnancy termination in the setting of IUFD. Multidisciplinary collaboration and the appropriate use of extracorporeal organ support may play a crucial role in optimizing maternal outcomes in such complex critical care scenarios.

## 1. Case Presentation

A 38‐year‐old pregnant woman (gravida 9, para 5) at 28 + 6 weeks of gestation presented with a 2‐day history of persistent abdominal pain and repeated vomiting. Owing to rapidly progressive metabolic deterioration, she was transferred to the intensive care unit (ICU) of Tianjin First Central Hospital for further evaluation and management.

The patient initially presented with abdominal pain and vomiting. At a local emergency department, obstetric ultrasonography demonstrated a viable intrauterine pregnancy; however, abdominal ultrasonography failed to adequately visualize the pancreas and therefore could not establish the diagnosis of acute pancreatitis (AP). After transfer to our emergency department, laboratory evaluation demonstrated profound metabolic derangements. Arterial blood gas analysis revealed severe metabolic acidosis (pH 7.05, bicarbonate 3.5 mmol/L, base excess −26.8 mmol/L). Blood glucose was 19.3 mmol/L, *β*‐hydroxybutyrate was 6.27 mmol/L, serum triglycerides were 100.22 mmol/L, total cholesterol was 27.14 mmol/L, serum amylase was 269 U/L, andlipase > 2000 U/L. Initial differential diagnoses included acute fatty liver of pregnancy (AFLP), Hemolysis, Elevated Liver enzymes, and Low Platelet count (HELLP) syndrome, biliary pancreatitis, and other causes of acute abdomen during pregnancy. During further evaluation, the patient′s condition progressively deteriorated. Repeat obstetric ultrasonography confirmed intrauterine fetal demise (IUFD), which was subsequently verified on follow‐up examination. Consequently, the therapeutic focus shifted from fetal–maternal management to prioritizing maternal stabilization.

**Figure 1:**
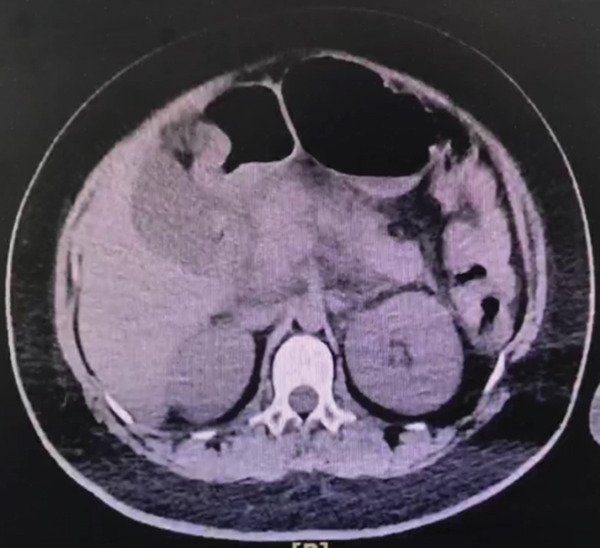
Abdominal CT showing pancreatic enlargement with marked peripancreatic inflammatory exudation, consistent with acute pancreatitis.

**Figure 2:**
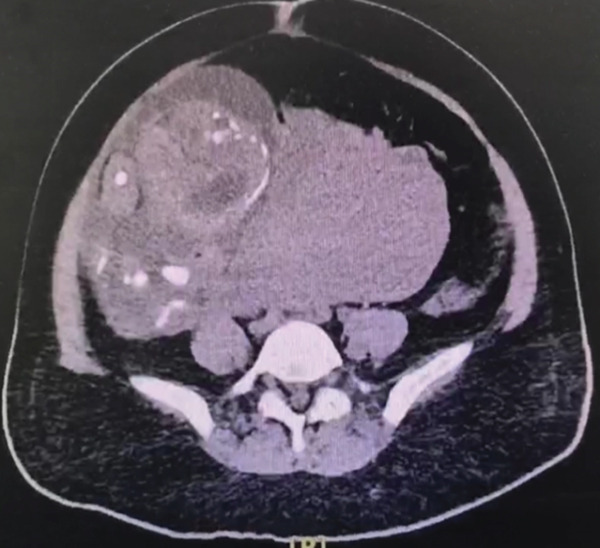
Abdominal CT demonstrating the intrauterine fetus, obtained during maternal evaluation for acute pancreatitis and metabolic derangements.

Abdominal computed tomography (CT) performed in the emergency department demonstrated pancreatic enlargement with peripancreatic inflammatory exudation, consistent with AP (Figures 1 and 2). Based on the combination of severe hypertriglyceridemia (100.22 mmol/L), markedly elevated serum lipase (> 2000 U/L), diabetic ketoacidosis (DKA), and characteristic imaging findings, the patient was diagnosed with hypertriglyceridemia‐induced acute pancreatitis (HTG‐AP) complicated by DKA. Before ICU admission, the patient had already received standard conservative management at both the referring hospital and our emergency department, including intravenous fluid resuscitation, insulin therapy, correction of electrolyte disturbances, and supportive care. However, serial arterial blood gas analyses demonstrated persistent and progressively worsening metabolic acidosis despite these conservative measures, with arterial pH declining from 7.27 to 7.05. Because severe metabolic acidosis remained refractory despite adequate conservative treatment, the patient was admitted to the ICU, where continuous renal replacement therapy (CRRT) was initiated to achieve rapid correction of metabolic acidosis and stabilization of metabolic homeostasis. Approximately 8 h after CRRT, arterial blood gas analysis demonstrated marked improvement in metabolic acidosis (pH 7.36, bicarbonate 21.6 mmol/L). However, serum triglyceride concentrations remained markedly elevated (82.45 mmol/L). Therefore, therapeutic plasma exchange (PE) using 2000 mL of fresh frozen plasma was performed to rapidly reduce the triglyceride burden.

**Figure 3:**
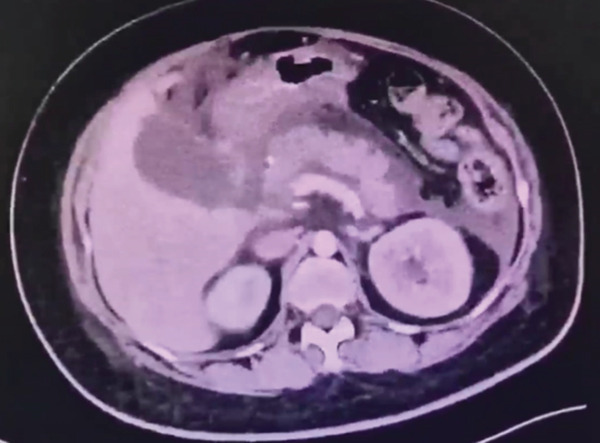
Repeat contrast‐enhanced abdominal CT showing increased peripancreatic inflammatory exudation and the presence of ascites, indicating progression of acute pancreatitis.

Despite correction of metabolic acidosis, the patient continued to experience persistent abdominal pain and progressive abdominal distension. Intra‐abdominal pressure reached 22 mmHg, consistent with intra‐abdominal hypertension. Repeat contrast‐enhanced abdominal CT demonstrated increased peripancreatic exudation and ascites (Figure 3), indicating progression of pancreatic inflammation.

Following multidisciplinary team (MDT) discussion involving specialists in critical care medicine, obstetrics, hepatobiliary surgery, endocrinology, gastroenterology, and interventional radiology, it was considered that immediate pregnancy termination before correction of the profound metabolic derangement would pose an unacceptably high maternal risk. Therefore, intensive metabolic stabilization was prioritized. Approximately 15 h after ICU admission, after severe metabolic acidosis had been corrected by CRRT and the triglyceride burden had been partially reduced following PE, emergency cesarean delivery was undertaken in view of persistent intra‐abdominal hypertension, radiological progression of pancreatitis, and confirmed IUFD.

**Figure 4:**
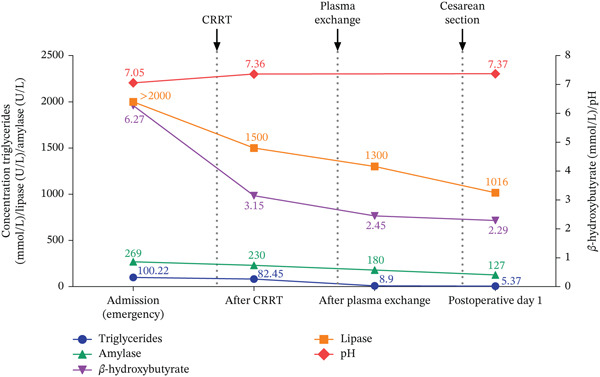
Dynamic changes in key laboratory parameters and major interventions.

Postoperatively, the patient was transferred back to the ICU for ongoing comprehensive critical care management. Supportive treatment, including glycemic control, correction of metabolic abnormalities, and lipid‐lowering therapy, was continued. On postoperative day 1, metabolic acidosis had resolved, and laboratory parameters showed marked improvement, with serum lipase decreasing to 1016 U/L, *β*‐hydroxybutyrate to 2.29 mmol/L, triglycerides to 5.37 mmol/L, and total cholesterol to 5.91 mmol/L. The patient′s clinical condition steadily improved, and she was subsequently transferred out of the ICU and discharged in good condition. The overall clinical course and the temporal changes in key laboratory parameters in relation to major therapeutic interventions are summarized in Figure 4.

## 2. Discussion

### 2.1. Pathophysiological Mechanisms of HTG‐AP Complicated by DKA During Pregnancy

Physiological changes in lipid metabolism occur during pregnancy under the influence of multiple hormones, characterized by increased hepatic lipoprotein synthesis and relatively reduced lipoprotein lipase activity, leading to elevated serum triglyceride levels [1]. When underlying disorders of glucose and lipid metabolism coexist, triglyceride concentrations may further increase and progress to pathological hypertriglyceridemia, substantially raising the risk of AP.

In the setting of DKA, insulin deficiency promotes lipolysis and hepatic very‐low‐density lipoprotein production, thereby exacerbating hypertriglyceridemia [2]. Excess triglyceride‐rich chylomicrons may accumulate within the pancreatic microcirculation, resulting in local ischemia and free fatty acid–mediated cytotoxic injury, which can trigger or aggravate pancreatic inflammation [3]. Conversely, the systemic inflammatory response and stress associated with AP may further worsen glycemic control, creating a vicious cycle in which hypertriglyceridemia, AP, and DKA mutually reinforce one another.

Therefore, HTG‐AP complicated by DKA during pregnancy should not be regarded as a consequence of a single etiological factor but rather as the result of multiple metabolic disturbances interacting within the unique physiological context of pregnancy. This case illustrates how these metabolic pathways may rapidly converge and progress during pregnancy, underscoring the importance of early recognition and comprehensive intervention to interrupt disease escalation.

### 2.2. Diagnostic Challenges of HTG‐AP During Pregnancy

Early diagnosis of HTG‐AP during pregnancy is challenging because both clinical manifestations and laboratory findings may be influenced by pregnancy‐related physiological changes and concurrent metabolic derangements. In the present case, abdominal pain was the initial presenting symptom; however, several pregnancy‐related conditions needed to be considered in the differential diagnosis, including AFLP, HELLP syndrome, biliary pancreatitis, and other causes of acute abdomen during pregnancy. In addition, initial abdominal ultrasonography failed to adequately visualize the pancreas, further delaying the diagnosis of AP.

In addition, the coexistence of DKA and extreme hypertriglyceridemia increased diagnostic complexity. Severe lipemia may interfere with laboratory assays, leading to underestimation of pancreatic enzyme levels and resulting in normal or only mildly elevated serum amylase or lipase values [4]. Meanwhile, DKA itself may be associated with elevated pancreatic enzymes in the absence of pancreatitis [5], further confounding differential diagnosis.

In the present case, abdominal CT was performed after IUFD had been confirmed. CT rapidly demonstrated characteristic findings of AP and provided important information regarding the extent of pancreatic inflammation and subsequent disease progression, thereby facilitating critical care management and multidisciplinary decision‐making. Accordingly, clinicians should maintain a high index of suspicion for HTG‐AP in pregnant patients presenting with abdominal pain accompanied by DKA or unexplained severe metabolic acidosis, particularly in the presence of marked hypertriglyceridemia, and pursue timely multidisciplinary evaluation.

### 2.3. Clinical Decision‐Making Regarding the Timing of Pregnancy Termination in the Setting of IUFD

The timing of pregnancy termination represents a pivotal clinical decision when HTG‐AP is complicated by severe metabolic derangement during pregnancy. Pregnancy itself imposes an increased metabolic burden and may amplify systemic inflammatory responses, whereas emergency surgical intervention in the context of profound metabolic instability may substantially increase perioperative risk. In pregnant patients with HTG‐AP, decisions regarding the timing of pregnancy termination should be guided not only by obstetric indications but also by the severity of pancreatitis, maternal metabolic stability, and the overall risk of perioperative deterioration.

In the present case, repeat ultrasonography confirmed IUFD and fetal benefit was no longer present, shifting the therapeutic goal toward prioritization of maternal safety [6]. Pregnancy termination itself does not constitute a specific treatment for AP; therefore, stabilization of maternal physiology should remain the primary therapeutic objective before surgery whenever clinically feasible. However, IUFD does not equate to an automatic indication for immediate pregnancy termination. Following multidisciplinary discussion, intensive supportive care and metabolic correction were initiated in the ICU prior to surgical intervention. Cesarean delivery was performed only after maternal metabolic status had been relatively stabilized, thereby reducing operative risk while facilitating continuation of definitive multidisciplinary management.

This staged decision‐making strategy highlights that, in cases of IUFD complicated by severe metabolic disturbances, the necessity of pregnancy termination may be clear, but the optimal timing should be individualized based on the severity of pancreatitis, maternal metabolic status, obstetric indications, perioperative risk, and available critical care support.

### 2.4. The Role of PE and CRRT in Pregnancy‐Related Metabolic Crisis

The cornerstone of treatment for HTG‐AP complicated by DKA during pregnancy is rapid correction of metabolic derangements to interrupt the underlying vicious cycle. Therapeutic PE enables direct removal of triglyceride‐rich plasma, achieving rapid lipid reduction and is considered a potential option for patients with extreme hypertriglyceridemia and severe complications [7]; however, its indications should be carefully selected.

In this case, CRRT primarily served as a metabolic support modality to correct refractory metabolic acidosis and stabilize the internal environment, functioning as a bridging strategy that allowed time for further interventions, including surgery [8]. Both PE and CRRT should be regarded as components of a comprehensive treatment approach rather than standalone therapies, and their application should be individualized based on close monitoring and multidisciplinary assessment.

This case suggests that, in complex clinical scenarios involving pregnancy‐related HTG‐AP and severe metabolic crisis, judicious integration of extracorporeal support therapies may improve short‐term metabolic control and reduce the risk of further clinical deterioration.

### 2.5. Prognosis of Pregnancy‐Associated HTG‐AP

HTG‐AP during pregnancy is generally associated with a higher risk of severe disease than other etiologies of AP because patients frequently present with profound metabolic disturbances, multiorgan dysfunction, and systemic inflammatory responses. Early recognition and aggressive multidisciplinary management are therefore essential to improve maternal outcomes [4].

Biliary pancreatitis remains the most common etiology of AP during pregnancy, whereas HTG‐AP is more frequently associated with severe metabolic derangements and may require intensive care support [9]. Hyperparathyroidism‐induced AP is another uncommon etiology during pregnancy. Unlike HTG‐AP, management primarily focuses on correction of hypercalcemia and definitive treatment of hyperparathyroidism. Therefore, prognosis largely depends on timely diagnosis and definitive treatment of the underlying endocrine disorder [10].

In the present case, despite the coexistence of severe metabolic acidosis, DKA, extreme hypertriglyceridemia, and IUFD, early intensive care support, CRRT, PE, and multidisciplinary decision‐making contributed to favorable maternal recovery.

## 3. Conclusion

HTG‐AP complicated by DKA during pregnancy is a rare but rapidly progressive metabolic emergency, and its clinical manifestations may be obscured by pregnancy‐related symptoms and profound metabolic disturbances. Early recognition is therefore essential. In the setting of IUFD, pregnancy termination should not be viewed as a single time‐point decision but rather as an individualized process guided by multidisciplinary collaboration and intensive supportive care. PE and CRRT may serve as valuable adjunctive strategies to stabilize metabolic status and create a therapeutic window for critical interventions. This case provides practical insight into the management of similarly complex clinical situations.

## Author Contributions

Yingshan Hu conceived the study, collected the clinical data, and drafted the manuscript. Yue Mao participated in data collection and literature review. Hongmei Gao supervised the study and critically revised the manuscript.

## Funding

This study was supported by the Tianjin Key Medical Discipline (Specialty) Construction Project (TJYXZDXK‐3‐023C), Tianjin Health Science and Technology Project (TJWJ2022XK023), Hebei Provincial Administration of Traditional Chinese Medicine Scientific Research Programme Project (T2025009), and Tianjin Health Science and Technology Project (TJWJ2025XK005).

## Disclosure

All authors read and approved the final manuscript. The lead author affirms that this manuscript is an honest, accurate, and transparent account of the case being reported; that no important aspects of the case have been omitted; and that any discrepancies from the case as originally planned have been explained. All scientific content, clinical interpretations, literature selection, and final revisions were independently performed and verified by the authors. The authors take full responsibility for the content of the manuscript.

## Consent

Written informed consent was obtained from the patient for publication of this case report and any accompanying images.

## Conflicts of Interest

The authors declare no conflicts of interest.

## Data Availability

The data that support the findings of this study are available from the corresponding author upon reasonable request.
